# Immune implication of an autophagy-related prognostic signature in uveal melanoma

**DOI:** 10.1042/BSR20211098

**Published:** 2021-08-20

**Authors:** Samuel Chuah, Valerie Chew

**Affiliations:** Translational Immunology Institute (TII), SingHealth-DukeNUS Academic Medical Centre, Singapore

**Keywords:** autophagy, immune microenvironment, Prognostic signature, uveal melanoma

## Abstract

Uveal Melanoma (UM) is a rare cancer deriving from melanocytes within the uvea. It has a high rate of metastasis, especially to the liver, and a poor prognosis thereafter. Autophagy, an intracellular programmed digestive process, has been associated with the development and progression of cancers, with controversial pro- and anti-tumour roles. Although previous studies have been conducted on autophagy-related genes (ARGs) in various cancer types, its role in UM requires a deeper understanding for improved diagnosis and development of novel therapeutics. In the present study, Zheng et al. used univariate Cox regression followed by least absolute shrinkage and selection operator (Lasso) regression to identify a robust 9-ARG signature prognostic of survival in a total of 230 patients with UM. The authors used the Cancer Genome Atlas (TCGA) UM cohort as a training cohort (*n*=80) to identify the signature and validated it in another four independent cohorts of 150 UM patients from the Gene Expression Omnibus (GEO) repository (GSE22138, GSE27831, GSE44295 and GSE84976). This 9-ARG signature was also significantly associated with the enrichment of cancer hallmarks, including angiogenesis, IL6-KJAK-STAT3 signalling, reactive oxygen species pathway and oxidative phosphorylation. More importantly, this signature is associated with immune-related functional pathways and immune cell infiltration. Thus, this 9-ARG signature predicts prognosis and provides deeper insights into the immune mechanisms in UM, with potential implications for future immunotherapy.

## Introduction

Uveal melanoma (UM) is a relatively rare but deadly subtype of melanoma that occurs at the eye, usually involving the iris, ciliary body or choroid (together known as the uvea) [1]. The tumours are known to derive from the melanocytes that reside within the uvea, and typically manifest with colorization and pain to the eye as well as vision impairment. UM has a high metastasis rate, particularly to the liver, and an overall poor prognosis where approximately 50% of the patients develop metastasis within 10 years of diagnosis with a survival of merely 6–12 months thereafter [2]. Due to the lack of effective treatment options, understanding the disease and identifying novel therapies remain a medical unmet need [3,4].

Autophagy, on the other hand, is a programmed intracellular degradative or digestive process which takes place under stressful conditions, including during the development and progression of cancers [5,6]. It plays a rather controversial role in both tumorigenesis and tumour-suppression; on one hand promoting the survival of tumour cells under metabolic stress, and on the other, inhibiting chronic inflammation and tumourigenesis [7]. Paradoxically, despite evidence that autophagy supports cancer cell survival [8], several autophagy-related genes (ARGs) such as Atg16 and p62 have been known to mitigate the resulting cellular damage from metabolic stress, thereby limiting inflammation and subsequent tumorigenesis [9,10]. Therefore, it is believed that different ARGs play distinct roles and also at different timeline along tumour development and progression.

In recent years, several ARGs were also found to be associated with the survival profile of patients with cancers such as in hepatocellular carcinoma [11], glioblastoma [12], colorectal cancer [13] and breast cancer [14]. Despite earlier efforts in understanding the roles of autophagy and UM [15,16], meta-analysis and data mining for ARGs in UM remains an important effort to unravel the relationship between ARGs in UM in order to achieve a deeper understanding of its roles in UM for either disease prognosis or potential identification of novel therapeutics.

The present study by Zheng et al. [17] performed data mining from five publicly available and independent UM cohorts and established a 9-ARG signature predictive of disease prognosis. These ARGs were also associated with unique intratumoural immune features which offered novel insights in understanding this rare malignancy.

## Identification of an ARGs signature predictive of UM prognosis

Zheng et al. [17] first obtained bulk RNAseq data from five UM cohorts: The Cancer Genome Atlas (TCGA) cohort, and four others from the Gene Expression Omnibus (GEO) repository (GSE22138, GSE27831, GSE44295 and GSE84976), and identified 423 ARGs which were expressed across all the cohorts. Using the TCGA cohort (*n*=80) as the training cohort, univariate Cox regression analysis narrowed this list down to 133 ARGs, which were associated with overall survival (OS). From these ARGs, a final prognostic signature of 9-ARGs was then established using Lasso Cox regression. The coefficient of each of these 9-ARG was then used to compute a risk score for each patient, who was then classified into low or high risk groups based on the median risk score of the cohort. Survival analysis based on this stratification showed that the 9-ARG signature successfully predicted survival rate in the TCGA cohort, and also showed correlations with other clinical and molecular features of UM patients such as loss of chromosome (Chr) 3, 6 and 8q, tumour thickness, age and clinical stage.

Extensive validation of the 9-ARG signature was then carried out in four other independent GEO cohorts (GSE22138, GSE27831, GSE44295 and GSE84976) of UM patients (total *n*=150). These cohorts were similarly stratified in to low or high risk, based on the median risk scores, and it was found that OS and disease-free survival were well-predicted by this 9-ARG signature. Furthermore, patients from the TCGA cohort were further stratified into low or high expression of each individual gene from the 9-ARG signature for survival analysis, where high expression of *IKBKE, BNIP1, ITGA6* and *FKBP1A* predicted worse OS, while high expression of *DLC1, PRKCD, GABARAPL1, LMCD1* and *TUSC1* predicted better OS. Among these genes, higher expression of IKBKE was previously shown to be associated with disease progression and metastasis in various cancers [18] while ITGA6 was found to play an important role in invasion and metastasis in breast cancer and was associated with shorter OS [19], consistent with their link to worser clinical outcome in UM. On the other hand, among the genes identified to be associated with better outcome in UM, DLC-1 was previously found to suppress progression and autophagy in hepatocellular carcinoma [20], and higher expression of GABARAPL1 was associated with a lower risk of metastasis in breast cancer [21]. These studies provide insights into how the expression of these genes might contribute to disease outcome in UM.

Finally, the ability of the 9-ARG signature to independently predict prognosis was tested with multivariate Cox regression against other common clinical factors. It was found that the stage of metastasis and the 9-ARG signature both possessed independent prognostic capabilities. Indeed, metastatic disease in UM, while rare, has previously been found to be associated with dismal survival rates [22]. The predictive ability of this signature was however independent of prior cancer therapies received by the UM patients in the TCGA cohort. The conclusion was that this 9-ARG signature is a robust risk factor which could predict survival outcome in UM patients, regardless of other potential confounding factors such as gender, age, stage and prior therapies received.

In fact, various previous studies in UM have focused on ARGs (either singly or as a signature) and their associations with prognosis [15,23,24]. In particular, the study by Cui et al. identified a long non-coding RNA autophagy-related signature predictive of disease outcome in UM [23]. However, amongst all these studies, the approach by Zheng et al. is unique as it builds the ARG prognostic signature in an unbiased manner from a collection of known transcriptomic ARGs, without any prior classification or risk-based segmentation of the cohorts before gene selection,.

## Functional and immunological implications of ARGs in UM

Finally, the authors investigated the differentially-enriched genes (DEGs) between low and high risk UM patients. Gene set enrichment analysis (GSEA) found that the tumour hallmarks of angiogenesis, IL6-JAK-STAT3 signalling, reactive oxygen species pathway and oxidative phosphorylation were enriched in high risk UM patients, consistent with disease progression. Among these tumour hallmarks, the IL6-JAK-STAT3 pathway has been well-described to promote cancer progression as well as immunosuppression in an autocrine manner [25] (Figure 2). Furthermore, the authors also found that genes involved in functions related to immune activation, differentiation, receptor activity/signalling and cytotoxicity were differentially-enriched between the two groups, which prompted them to examine the immune microenvironment.

Using the CIBERSORT algorithm and other immune-related scoring algorithms, they inferred that UM patients belonging to the higher risk group displayed greater immune and stromal cell infiltration, especially by CD8 T cells, activated memory CD4 T cells and T follicular helper cells. This finding is seemingly counterintuitive, since in many solid cancers, greater immune cell infiltration, especially T cells, is usually associated with good prognosis [26]. Nevertheless, in UM, a number of previous studies have also found that UM with poorer prognosis are indeed more highly infiltrated with immune cells, and that they display potentially immunosuppressive phenotypes [27,28]. One potential explanation is that the human eye is known to be an immune-privileged organ and therefore greater immune infiltration such as that induced by genetic abnormalities (e.g. monosomy 3 and gain of chromosome 8q) could result in a higher risk in UM [22]. Thus, this 9-ARG signature shows its utility not just in predicting the OS of UM patients, but also in understanding of the potential immune mechanisms driving disease outcome.

## Conclusion

**Figure 1:**
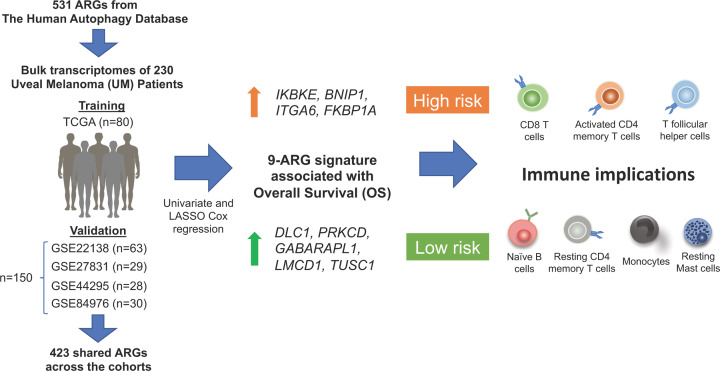
Identification and validation of autophagy-related genes (ARGs) signature in uveal melanoma (UM) Schematic illustration of screening and identification of 9-ARG signature from TCGA training cohort (*n*=80) and four GEO validation cohorts (*n*=150). Expression of different ARGs and immune infiltrates associated with high versus low risk UM was also demonstrated.

In recent years, we observed the increasing trend of omics data mining using advanced bioinformatics tools to identify prognostic biomarkers for cancers. In the current study, Zheng et al. [17] combined univariate and Lasso Cox regression models to develop a prognostic 9-ARG signature which segregated a total of 230 UM patients from five independent public cohorts according to their distinct clinical outcomes (Figure 1). The nine prognostic ARGs: *IKBKE, BNIP1, ITGA6, FKBP1A, DLC1, PRKCD, GABARAPL1, LMCD1* and *TUSC1*, and their important roles in immune cell infiltration, provided valuable insights that could have future implications in immunotherapy for UM. However, further mechanistic validation using *in vitro* or *in vivo* systems will be necessary to strengthen the link between this ARG signature and disease outcome. Furthermore, the study should be expanded beyond its current scope to help in identifying key regulator(s) that could be harnessed as novel therapies for UM.

**Figure 2:**
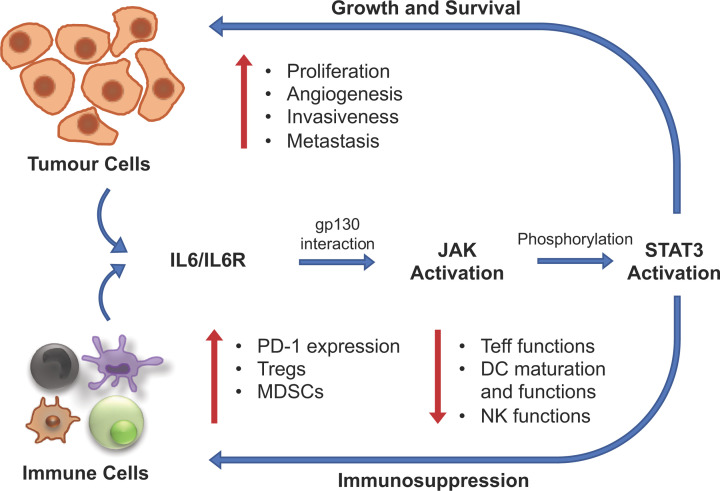
Potential association of the IL6/JAK/STAT3 pathway in promoting tumour cell growth and immunosuppression in UM IL6 is secreted by both tumour cells and immune cells within the tumour microenvironment and binds to its receptor, IL6R. Upon interaction with the IL6R subunit β (gp130), also found on both tumour and immune cells, JAK enzymes are activated which can then phosphorylate STAT3, leading to its dimerization and activation. Translocation of the STAT3 dimer to the nucleus promotes the expression of target genes, leading to the promotion of tumour growth and immunosuppression.

## Abbreviations

ARG: autophagy-related gene
DEG: differentially expressed gene
GEO: Gene Expression Omnibus
OS: overall survival
TCGA: The Cancer Genome Atlas
UM: uveal melanoma
